# Reduced calorie diet combined with NNMT inhibition establishes a distinct microbiome in DIO mice

**DOI:** 10.1038/s41598-021-03670-5

**Published:** 2022-01-10

**Authors:** Andrea Dimet-Wiley, Qinglong Wu, Jerrin T. Wiley, Aditya Eswar, Harshini Neelakantan, Tor Savidge, Stan Watowich

**Affiliations:** 1 Department of Biochemistry and Molecular Biology, University of Texas Medical Branch, 301 University Boulevard, Galveston, TX USA; 2 Department of Pathology and Immunology, Baylor College of Medicine, Houston, TX USA; 3 Depatment of Computer Science, University of Houston, Houston, TX USA; 4 New York University Stern School of Business, New York City, NY USA; 5 Ridgeline Therapeutics, Houston, TX USA

**Keywords:** Bioinformatics, Diagnostic markers, Microbiome, Obesity

## Abstract

Treatment with a nicotinamide N-methyltransferase inhibitor (NNMTi; 5-amino-1-methylquinolinium) combined with low-fat diet (LD) promoted dramatic whole-body adiposity and weight loss in diet-induced obese (DIO) mice, rapidly normalizing these measures to age-matched lean animals, while LD switch alone was unable to restore these measures to age-matched controls in the same time frame. Since mouse microbiome profiles often highly correlate with body weight and fat composition, this study was designed to test whether the cecal microbiomes of DIO mice treated with NNMTi and LD were comparable to the microbiomes of age-matched lean counterparts and distinct from microbiomes of DIO mice maintained on a high-fat Western diet (WD) or subjected to LD switch alone. There were minimal microbiome differences between lean and obese controls, suggesting that diet composition and adiposity had limited effects. However, DIO mice switched from an obesity-promoting WD to an LD (regardless of treatment status) displayed several genera and phyla differences compared to obese and lean controls. While alpha diversity measures did not significantly differ between groups, beta diversity principal coordinates analyses suggested that mice from the same treatment group were the most similar. K-means clustering analysis of amplicon sequence variants by animal demonstrated that NNMTi-treated DIO mice switched to LD had a distinct microbiome pattern that was highlighted by decreased *Erysipelatoclostridium* and increased *Lactobacillus* relative abundances compared to vehicle counterparts; these genera are tied to body weight and metabolic regulation. Additionally, *Parasutterella* relative abundance, which was increased in both the vehicle- and NNMTi-treated LD-switched groups relative to the controls, significantly correlated with several adipose tissue metabolites’ abundances. Collectively, these results provide a novel foundation for future investigations.

## Introduction

Obesity remains a poorly-controlled, global public health problem^1–3^. First-line obesity treatments include lifestyle intervention (e.g., low-calorie diets) and behavioral modification therapies^4,5^ since approved obesity medications have limited efficacy and commonly have side effects^6^. A novel target for next-generation obesity medications is nicotinamide N-methyltransferase (NNMT)^7–11^, an enzyme central to cellular metabolism and energy homeostasis^12^. The use of NNMT inhibitors as a potential obesity treatment has been investigated preclinically^8,9,13^, and observational clinical data suggests that increased NNMT expression and activity correlate with increased incidence of Type 2 diabetes (T2D) and risk of obesity, respectively^10,11^. In proof-of-concept studies, NNMT inhibitors (NNMTis) have demonstrated promise as potential treatments for obesity and obesity-related metabolic comorbidities (e.g., T2D, fatty liver)^8,9,13^. However, the role of the microbiome in NNMTi-mediated weight loss is yet to be elucidated.

Understanding how the microbiome is involved in establishing, maintaining, and reversing obesity has been an active area of research^14–16^, and has included studies investigating links between the microbiome and obesity-related comorbidities such as non-alcoholic fatty liver disease (NAFLD) and non-alcoholic steatohepatitis (NASH)^17,18^. For example, individuals with NAFLD exhibit increased intestinal permeability and prevalence of small intestine bacterial overgrowth, and these factors are associated with the severity of hepatic steatosis^19^. Additionally, recent studies have identified bacterial DNA in human and mouse adipose tissues^20,21^. Increased intestinal permeability is facilitated by a high-fat diet (HFD)^22^, and may allow for bacterial translocation from the gut to adipose tissue^23^. Since adipose tissue is responsive to endotoxins, bacterial translocation may provide a mechanism whereby the gut microbiome can modulate the adipose metabolome (for review, Kruis et al., 2014^23^) and consequently exacerbate obesity and its related comorbidities.

We recently reported that NNMTi treatment combined with a low-fat, or lean, diet (LD) returned systemic adiposity and liver pathologies of obese animals to the adiposity and liver pathology levels of LD controls. LD alone, however, failed to improve obesity-linked liver pathologies^13^. Given that both diet composition and adiposity can each substantially impact the gut microflora in obese mice^24^, we conducted a secondary endpoint study using animals given the same series of diets to test the hypothesis that NNMTi treatment combined with LD, but not LD alone, shifts the cecal microbiome profile of obese animals to be similar to age-matched LD controls that were never given a high-fat diet. This study used the NNMTi 5-amino-1-methylquinolinium (5A-M1Q), since our lab has set precedence using this probe molecule for proof-of-concept studies^8^.

Additionally, the microbiomes in these treatment groups were compared to both age-matched lean and obese controls. Moreover, given the potential links between adipose tissue metabolism and the gut microbiome, correlations between the cecal microbiome and adipose tissue metabolites were examined. Collectively, this study provides a solid foundation for future exploration into the impact of NNMTi-and diet-mediated changes to the gut microbiome in obese and lean populations, as well as future targets to investigate mechanistically with NNMTi treatment.

## Methods

### Animals

Animal experiments were performed with adherence to all national and local guidelines and regulations, the Guide for the Care and Use of Laboratory Animals^25^, and the ARRIVE guidelines, and with the approval of the Institutional Animal Care and Use Committee at The University of Texas Medical Branch (UTMB). Male, 18-week-old C57BL/6J mice, maintained on a 60% high-fat diet (HFD; 60 kcal% fat, 20 kcal% carbohydrate, 20 kcal% protein, Research Diets, Inc. OpenSource Diets formula D12492; DIO mice, JAX cat no. 380050) or a low-fat (lean) diet (LD; 10 kcal% fat, 70 kcal% carbohydrate, 20 kcal% protein, Research Diets, Inc. OpenSource Diets formula D12450B; DIO control mice, JAX cat no. 380056) from 6 to 18 weeks of age, and co-housed with counterparts on the same diet, were purchased from The Jackson Laboratory (Bar Harbor, ME, USA). Upon arrival at UTMB, the 18-week-old mice were single-housed and provided ad libitum access to water and a Western diet (WD; 45 kcal% fat, 35 kcal% carbohydrate, 20 kcal% protein, Research Diets, Inc. OpenSource Diets formula D12451) or, if they were previously on an LD, they were maintained continued on LD (termed lean control mice). Mice were maintained in one controlled colony room, kept at 21–23 °C and 45–50% humidity with a 12-h light–dark cycle (lights on 6 a.m.–6 p.m. Central Standard Time [CST]). Cages were exchanged for new sanitized cages filled with irradiated bedding every 2 weeks. Water was changed weekly. Up to two pieces of enrichment were included in each cage.

**Figure 1:**
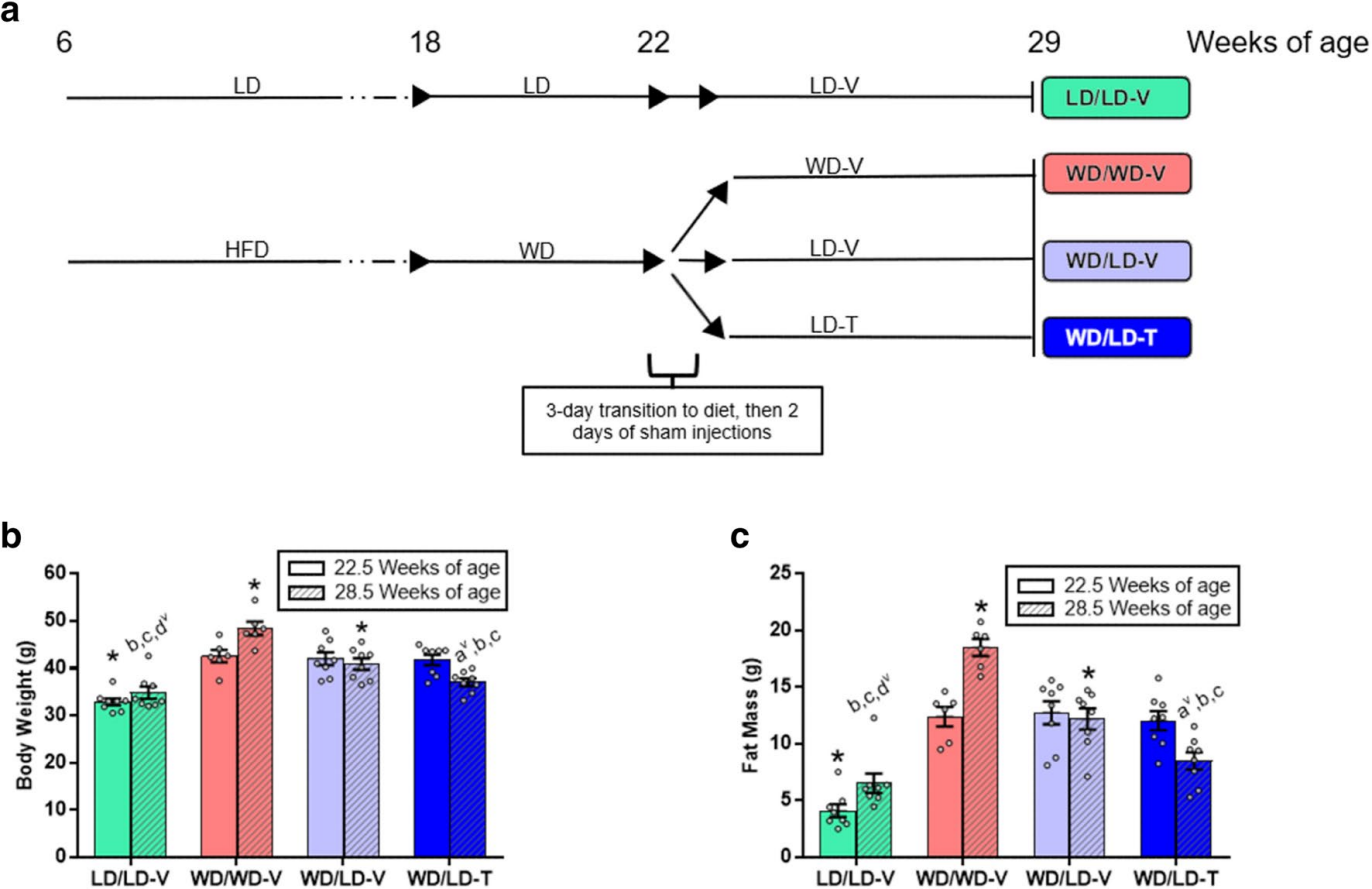
Diet and NNMTi treatment status substantially altered body weight and fat mass. **a** After weaning, mice were placed on a high-fat diet (HFD, 60% fat) for 12 weeks, transitioned to Western diet (WD, 45% fat) for 4 weeks, and then randomized to WD or to lean diet (LD, 10% fat) and vehicle (-V) or NNMTi treatment (-T) with 5-amino-1-methylquinolinium (5A-1MQ) for approximately 7 weeks prior to study termination and cecal sample collection; a control group remained on LD throughout the study. **b,c** Body weight and fat mass were significantly different between the diet-induced obesity model and the lean control group at study start (22.5 weeks of age); at the end of the study, body weight and fat mass were lower in the WD/LD-V group than the WD/WD-V control group, and in the NNMTi-treated group these measures were even lower and statistically indistinguishable from the LD/LD-V control group when a correction for multiple comparisons was not run (n = 6–8). Graphs depict mean + /− SEM; unless specified, multiple comparisons were significant before and after FDR correction (*p* < 0.05 and q < 0.05); further statistical details can be found in Supplementary Table S2. *, significantly different from all other groups for that study day; *a*, significantly different from the lean control group (LD/LD-V); *b*, significantly different from the obese control group (WD/WD-V); *c*, significantly different from vehicle-treated mice switched from WD to LD (WD/LD-V); *d*, significantly different from 5A-1MQ-treated mice switched from WD to LD (WD/LD-T); ^v^, *p* > 0.05 but q < 0.05.

The DIO mice were randomized into three treatment groups balanced by baseline body weight and body composition (fat and lean mass) measures. Two of the three groups were transitioned from WD to LD for 5 days before the start of the study. All mice received 2 days of 10 mL/kg body weight sterile saline subcutaneous injections before beginning the treatment phase to allow for acclimation to the handling and stress associated with injections. Control groups of mice were maintained on LD or WD and given sham (10 mL/kg body weight sterile saline) injections throughout the study (termed LD/LD-V, or LD control, and WD/WD-V, or WD control, groups; n = 6–8 mice/group). One group of mice that were switched from WD to LD received these same sterile saline injections for the remainder of the study (termed the WD/LD-V group; n = 8), while a second group received 5A-1MQ treatment at 32 mg/kg of active pharmaceutical ingredient (injections of 4 mg 5A-1MQ monochloride salt/mL sterile saline dosed at 10 mL/kg body weight) after being switched from WD to LD (termed the WD/LD-T group; n = 8). Subcutaneous injection was chosen as 5A-1MQ specifically has poor oral bioavailability in mice, a phenomenon that does not occur in rats^26^. This dose was chosen based on a previous dose-escalation study and subsequent work demonstrating its efficacy to drive weight loss in a short period of time^8^. Study design, and diet and treatment groups are diagrammed in Fig. 1a. Daily doses were given between approximately 4–6 PM CST. Food intake and body weight were measured twice weekly. Body composition was determined weekly with an EchoMRI 4in1-500 Body Composition Analyzer (EchoMRI Whole Body Composition Analyzer; EchoMRI LLC, Houston, TX, USA). Injection volumes were calculated using the most recently recorded animal weight and adjusted weekly; for more details, see Sampson et al., 2021^13^. Mice were euthanized in a non-fasted state over 2 days during their sleep cycle; six animals from the LD control, WD control, and WD/LD-V groups and five animals from the WD/LD-T group were euthanized the first day. The remaining two LD/LD-V, two WD/LD-V, and three WD/LD-T mice were euthanized 2 days later. One individual per group was euthanized before addressing the next animal in each group to render differences arising from the time of day at euthanization (e.g., cecal mass changes resulting from differences in time post-food consumption) comparable across all groups.

### Cecal samples

Cecal weight was measured since the weight of this portion of the intestine appears to be highly influenced by the microbiome (e.g., certain antibiotic treatments dramatically increase cecal weight^27–31^ but not the colon^27^). Cecal samples were collected at euthanization, then flash-frozen and stored at − 80 °C. DNA was not isolated from the first two samples collected in the LD/LD-V group, and the first sample collected in each of the other respective groups, due to the use of saline to flush the cecum (after weighing) during early collection (therefore, n = 25 cecal samples). BGI (Yantian District, Shenzhen, China), an established contract research organization, isolated DNA from the remaining cecal samples and generated a metagenomic 16s rDNA amplicon library. Fusion primers with dual-indexed PCR adapters were used to generate the construct library, and short fragments were removed with AMPure beads. DNA concentration was determined with a Qubit dsDNA BR Assay Kit (Thermo Fisher Scientific) and Qubit fluorometer (Thermo Fisher Scientific).

### Sequencing, data processing, and analysis parameters

The study's primary aim was to determine whether 5A-1MQ treatment in combination with a switch from WD to LD rendered the intestinal microbiome comparable to that of mice continuously maintained on LD, and distinct from that of DIO mice switched to LD but given saline injections or mice maintained continuously on WD. Microbiome 16S V1-V3 rRNA gene sequencing, alignment, and initial filtering were performed by BGI. Sequencing was performed using a HiSeq 2500 (Illumina), with a read length of 300 bp paired-end reads and at least 25,000 tags per sample. Reads with an average quality < 20 over a 25 bp sliding window were truncated (phred algorithm^32,33^), and the trimmed reads with < 75% of their original length were removed along with their respective pair. Additionally, reads contaminated by the adapter (reads with ≥ 15 bases overlapping between the reads and the adapter with up to 3 bases mismatching), reads with ambiguous bases, and reads with ≥ 10 identical consecutive bases were removed along with each of their respective paired reads. Cleaned reads were assigned to their corresponding samples by requiring 0 base mismatches to the barcode sequences, as determined by in-house (BGI) scripts. For paired-end reads, a consensus sequence was created using Fast Length Adjustment of SHort reads^34^ (v1.2.11) with minimal overlapping length (15 bp) and a mismatching ratio of the overlapped region ≤ 0.1; paired-end reads not meeting this criteria were removed. Combining the paired-end reads with the tags based on overlapping regions generated 3,360,747 tags identified in total for all samples, with an average of 134,429 tags/sample. Sequences with less than four consecutive bases matching the tags at the 3′ end and > 2 mismatches in the bases for the remaining part of the primer were removed, and this resulted in a total of 3,332,352 tags, averaging 133,294 tags/sample with an average length of 481 bp.

These sequences were then run through an R script (Supplementary Materials, bash_DADA2 + IDTAXA_workflow_Illumina-PairedEnd_V1V3.R) which trimmed reverse reads to 260 bp, removed forward reads exceeding a maximum expected error of three, and truncated reverse reads exceeding a maximum expected error of six. Filtered reads were then paired-end merged again, and chimeras and non-bacterial sequences were discarded from the data. Paired-end FASTQ files were then run through the DADA2 pipeline (v1.8) to generate an amplicon sequence variant (ASV) table and assign taxonomy. DECIPHER (v2.6.0) was used to align sequences, and FastTree (v2.1.3) was used to generate a phylogenetic tree.

Two analyses were performed to determine the species comprising the genera. The first method used a Basic Local Alignment Search Tool Nucleotide (BLASTn; v2.7.1; script in Supplementary Materials Scripts .zip file, BLASTn Query) search^35,36^; this assigned the best hit for each of the ASVs and then provided the percentage of identical matches between the sequence of interest and the top hit as well as an e-value (i.e., the number of expected hits with a similar quality that could be found by chance). Unfortunately, the e-value is affected by the database size and several gaps and mismatches could be present without dramatically influencing the e-value. Consequently, a second method, Bayesian Latent Class Analysis-based Taxonomic Classification (BLCA; Python v3; https://github.com/qunfengdong/BLCA; accessed November 25, 2020; script in Supplementary Materials Scripts .zip file, BLCA), was used that output bootstrap confidence intervals determined using Bayesian posterior probability and weighing the similarity between database hits relative to the query sequence. In this second approach, higher similarity resulted in greater weight and, consequently, greater contribution to taxonomic assignment. The species results of these two methods were cross-compared and a result was only considered definitive if it was identified using both methodologies.

The BIOM file output from the DADA2 pipeline and the DECIPHER-aligned FastTree phylogenetic tree were input into QIIME (v1.9.1) and the scripts in the Supplementary Materials (QIIME Scripts compilation) were used to establish the alpha diversity metrics of Chao1 richness estimate, Simpson’s evenness measure E, Shannon diversity index H, Simpson’s diversity index, and Faith’s phylogenetic diversity metric (whole tree) as well as the beta diversity measures of unweighted and weighted UniFrac analyses (the latter of which factors in ASV abundance^37^) and Bray–Curtis dissimilarity analysis. Principle coordinates analyses (PCoAs) were generated based on the distance and dissimilarity matrices.

From the ASV table (Supplementary Table S1), sums of ASVs within each respective phylum or genus for each sample were calculated to establish the abundance of each phylum or genus per sample. The sums of the number of unique ASVs per sample were compared across groups, with the unclassified sequences excluded for the observed ASVs; this was used to estimate richness. Relative abundance of each phylum or genus was calculated in each sample by dividing the abundance of the respective phylum or genus by the sum of the abundances of all phyla or genera for that sample, then multiplying by 100 to generate a percentage. K-means clustering was performed on the genera relative abundance data using the R script in the Supplementary Materials Scripts .zip file (KMScript), and clusters were plotted. The number of clusters was determined using the sum of squared estimate of errors curve.

### Statistics

Statistics were run using GraphPad Prism (v7.05) unless specified otherwise. For the relative abundance data, phyla and genera data were first transformed by adding 1e−13 to each sample. Subsequently, for these data as well as the alpha diversity analyses, each treatment group (within each phylum or genus for the relative abundance data) was analyzed for normal distribution (Shapiro–Wilk test) and the data across groups were tested for homoscedasticity (Brown–Forsythe test). Data were log_10_ transformed if they were non-normally distributed and/or heteroscedastic. Alpha diversity analyses did not require the initial 1e−13 transformation as all values were > 0. Data that remained non-normally distributed and/or heteroscedastic after log transformation were analyzed (not log-transformed, but still 1e−13-transformed) with a Kruskal–Wallis test, while normally distributed and homoscedastic data were analyzed with a one-way ANOVA, performed on the log-transformed data as necessary. Datasets that included a group demonstrating zero variance between samples were analyzed non-parametrically. Significant ANOVA/Kruskal–Wallis test results were then followed with post-hoc testing, which underwent the two-stage linear step-up procedure of Benjamini, Krieger and Yekutieli correction for multiple comparisons. Because a large number of comparisons were performed on the relative abundance data, main effect results from these analyses also underwent a Benjamini–Hochberg false discovery rate (FDR) correction for multiple comparisons, and instances where a result was significant after this correction have been duly noted. For the beta diversity analyses, an analysis of similarities (ANOSIM) was performed on each of the distance/dissimilarity matrices to establish whether observed clustering by treatment group was statistically significant.

In addition to the analyses on the relative abundance data mentioned above, taxa were summarized using the Python script labeled QIIME Scripts compilation in the Supplementary Materials Scripts .zip file. The tabular data were processed with the Huttenhower lab’s linear discriminant analysis (LDA) effect size (LEfSe) tool via the Galaxy module (v1.0)^38^. LEfSe analysis provided a secondary measure to establish differences between treatment groups in addition to the one-way ANOVA/Kruskal–Wallis tests performed on each genus/phylum. LEfSe is known to reduce false positives and can highlight biological consistency across subclasses, while the Kruskal–Wallis test used alone can achieve a lower false-negative rate compared to LEfSe^38^. For the LEfSe analysis, an alpha of 0.05 was set for the Kruskal–Wallis tests among classes, and per-sample normalization was performed to bring the sum of the values to 1 M. The log_10_ LDA score threshold was set to 2.0, and all classes were compared against all other classes for multi-class analysis.

### Development of a tool for correlational analyses

Since a secondary aim of this study was to examine potential relationships between the cecal microbiome and adipose tissue metabolome, a Python (v3.8) tool, which used modules from SciPy^39^, was created to expedite the correlational analyses between the cecal microbiome and the previously-published epididymal white adipose tissue (EWAT) metabolome^13^. The tool followed the decision tree in Supplementary Fig. S1. It used a Shapiro–Wilk test to assess normality and an F-test to assess homoscedasticity, and attempted log-transformation to resolve any lack thereof^40^. This novel tool, termed the Spearman’s-Pearson’s Decider, which expedited the processes needed to decide between a parametric or non-parametric analysis, is freely available at GitHub (https://github.com/JerrinWiley/Spearmans_Pearsons_Decider; script and ReadMe in Supplementary Materials Scripts .zip file under Spearmans_Pearsons_Decider and ReadMe for Spearmans_Pearsons_Decider, respectively). An assumption of parametric analyses that was not tested by the tool was a lack of outliers; this was not included in the tool since the definition of outliers is subjective. However, outliers were analyzed for these correlations using the interquartile range (IQR) method; quartiles were computed in Microsoft Excel and the upper range for outliers was set at quartile 3 + (1.5 × the IQR), and the lower range was set at quartile 1 − (1.5 × the IQR). Outliers were then removed, and the data were run through the Spearman’s-Pearson’s Decider again. Finally, the correlation coefficient for each combination of genus and metabolite with outliers was compared to the coefficient without outliers for the same combination; correlation coefficients that changed > 0.2 were noted as being influenced by the presence of outliers; however, these results were not excluded from corrections for multiple comparisons or the heat map generated with the data. Benjamini–Hochberg false-discovery rate corrections (set to 5%) were applied to the results with and without outliers individually.

For each analysis, the statistical test used and n for each group are found in Supplemental Tables 2, 3, or 5; all tests were two-tailed and α = 0.05 for each analysis. When comparisons were made, all relevant treatment groups were included.

## Results

### Body weight, fat mass, and cecal mass

As expected, obese mice (maintained on an HFD followed by WD) had significantly higher body weights than mice continuously maintained on LD. However, the body weight of 5A-1MQ-treated obese mice (WD/LD-T group), which was indistinguishable from the other DIO groups at study start, was indistinguishable from the lean control (LD/LD-V) group at study completion (results with FDR correction; study timeline, Fig. 1a; body weight results Fig. 1b, Supplementary Table S2). Fat mass followed the same pattern. All mice that were subjected to an HFD had a higher fat mass compared to the LD control group at the start of the study. Importantly, at study completion, 5A-1MQ-treated mice had a lower fat mass than the obese WD controls and their vehicle-treated counterparts and were not significantly different (with FDR correction) from the LD control mice (Fig. 1c; Supplementary Table S2). Cecal mass did not significantly differ between groups (Supplementary Table S2).

### Alpha diversity

**Figure 2:**
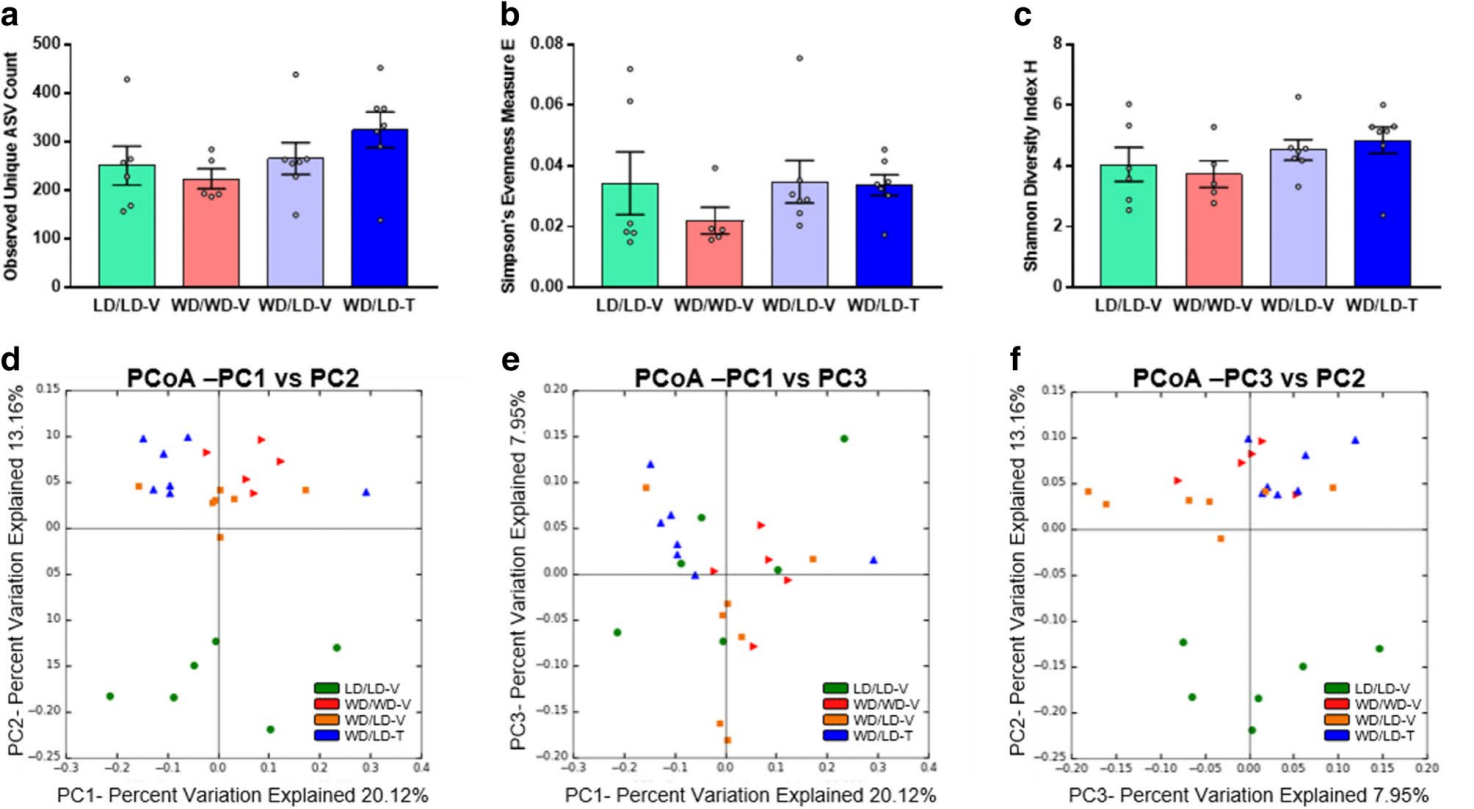
Alpha diversity measures did not differ significantly across treatment groups, and unweighted UniFrac beta diversity analysis exhibited partial clustering by treatment group. Observed unique ASV count (**a**), Simpson’s Evenness Measure E (**b**), and Shannon Diversity Index H (**c**) were all statistically indistinguishable between groups; graphs depict mean + /− SEM. Unweighted UniFrac analysis is presented as a PCoA (**d–f**) and exhibits distinct clustering, with a large separation of the LD control group from the other groups.

Several alpha diversity measures were analyzed since bacterial evenness and richness can be incorporated to varying degrees in different measures^41^. Bacterial richness measured by the unique ASV counts observed did not significantly differ between treatment groups (Fig. 2a). Additionally, no differences between treatment groups were identified using Simpson’s evenness measure E (Fig. 2b) or when richness and evenness were combined in the Shannon–Wiener Diversity Index H (Fig. 2c). No significant differences between treatment groups were observed using the Chao1 richness estimate^42^, which accounts for ASV rareness in the richness measure (Supplementary Fig. S2). Furthermore, there were no differences between treatment groups for Simpson’s Diversity Index (Supplementary Fig. S2) or phylogenetic diversity as measured by Faith’s formula applied to the whole phylogenetic tree (Supplementary Fig. S2). Although the mean values for each of the alpha diversity measures were often largest for the 5A-1MQ-treated group switched from WD to LD relative to all other groups (Fig. 2; Supplementary Fig. S2), this trend did not rise to the level of statistical significance.

### Beta diversity

Beta diversity was determined using the unweighted UniFrac, weighted UniFrac, and Bray–Curtis dissimilarity measures. PCoAs were generated using the distance matrices from unweighted (Fig. 2d-f) and weighted (Supplementary Fig. S2) UniFrac analyses and Bray–Curtis dissimilarity analysis (Supplementary Fig. S2). These graphs suggested that there may be, at least some degree of, clustering by treatment group. Consequently, ANOSIM testing was also performed, which showed that clustering by treatment group was significant in each of the diversity measures (unweighted UniFrac: R value = 0.369, *p* = 0.0010; weighted UniFrac: R value = 0.190, *p* = 0.0240; Bray–Curtis: R value = 0.165, *p* = 0.0060).

### Phyla

**Figure 3:**
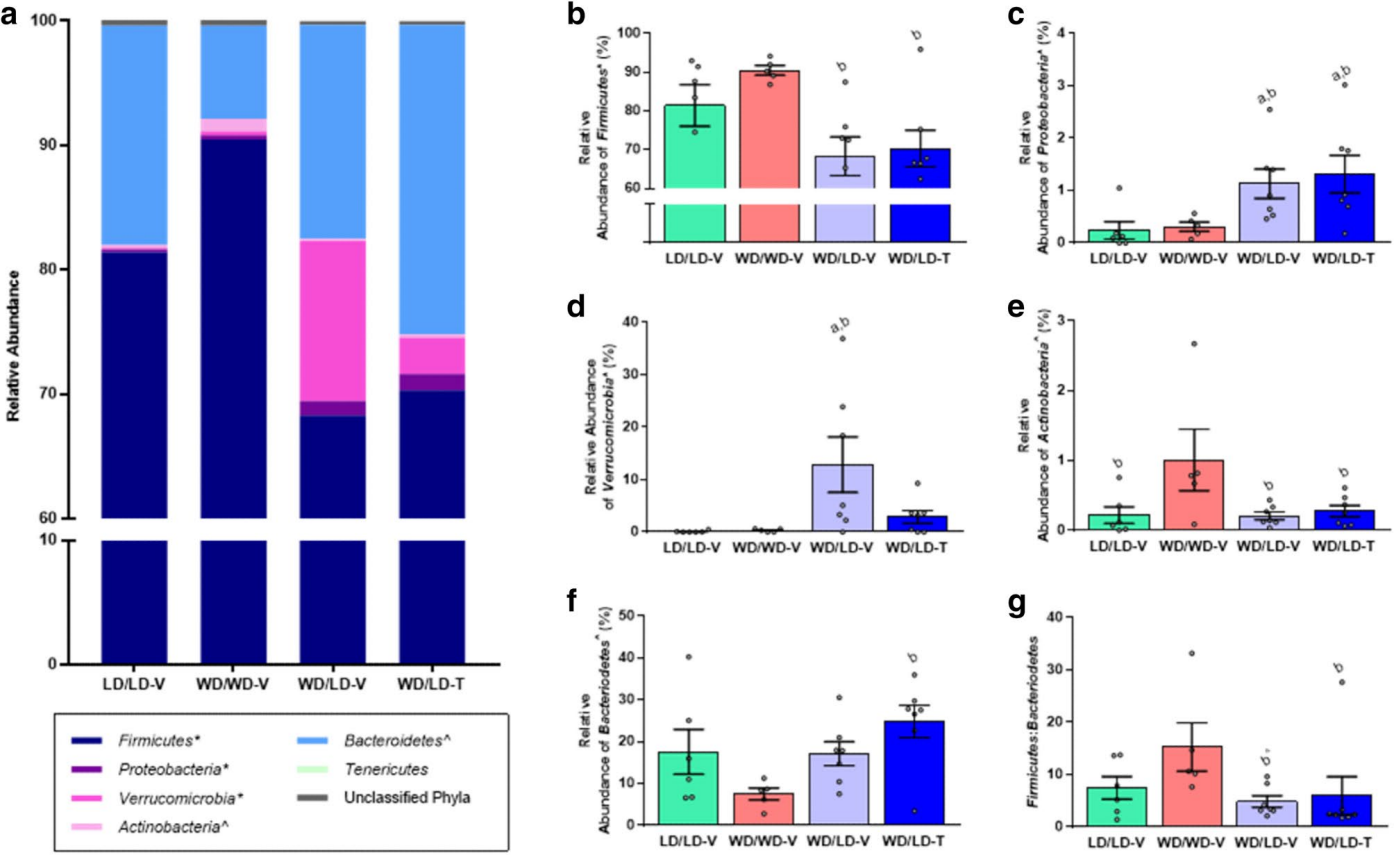
Microbial phyla exhibit dramatic shifts predominantly mediated by diet switch as opposed to diet composition. (**a**) Relative abundance for each of the phyla identified in the mouse cecal samples as well as the remaining sequences which could not be assigned (unclassified). (**b–d**) Three bacterial phyla demonstrated significant differences following Benjamini–Hochberg correction for multiple comparisons: *Firmicutes*, *Proteobacteria*, and *Verrucomicrobia.* (**e–f**) *Actinobacteria* and *Bacteroidetes* both demonstrated significant differences before (but not after) Benjamini–Hochberg correction. (**g**) The ratio of *Firmicutes:Bacteroidetes* was significantly lower in the WD/LD-T mice compared to those maintained on WD, and there was a trend for a similar effect in the WD/LD-V mice. Graphs depict mean + /− SEM; unless specified, multiple comparisons were significant before and after FDR correction (*p* < 0.05 and q < 0.05); further statistical details can be found in Supplementary Table S3. *a*, significantly different than the lean control group (LD/LD-V); *b*, significantly different than the obese control group (WD/WD-V); *^*, p < 0.05 but q > 0.05.

Six distinct phyla were identified in the cecal microbiomes, but not all were present in each sample. Sequences that could not be definitively assigned to a phylum were labeled “unclassified” (Fig. 3a; Supplementary Table S3). Of these seven groups, the *Firmicutes*, *Proteobacteria*, and *Verrucomicrobia* phyla demonstrated a significant main effect of treatment group on relative abundance after Benjamini–Hochberg correction for multiple comparisons, while *Bacteroidetes* and *Actinobacteria* demonstrated significant main effects of treatment group on relative abundance before Benjamini–Hochberg correction. Post-hoc comparisons showed that the *Firmicutes* phylum’s relative abundance was significantly higher in WD control mice compared to mice in the vehicle- and 5A-1MQ-treated groups switched from WD to LD (Fig. 3b). In contrast, *Proteobacteria* relative abundance was significantly higher in both the vehicle- and 5A-1MQ-treated groups switched from WD to LD, compared to both the LD and WD control groups (Fig. 3c). *Verrucomicrobia* relative abundance was significantly higher in vehicle-treated mice switched from WD to LD compared to both the LD and WD control groups (Fig. 3d). *Actinobacteria* showed a significantly greater relative abundance in WD control mice compared to all other groups (Fig. 3e). Finally, *Bacteroidetes* relative abundance was significantly increased in 5A-1MQ-treated mice switched from WD to LD relative to WD control mice (Fig. 3f), and its relative abundance was lowest in WD control mice compared to all other groups.

The ratio of *Firmicutes* to *Bacteroidetes* is a commonly-studied change with an increase often linked to HFD consumption/obesity in mice^43,44^ and humans^45,46^ (for review, see John and Mullin, 2016^47^). In the current study, the ratio of *Firmicutes* to *Bacteroidetes* was significantly higher in WD control mice compared to both groups of mice transitioned from WD to LD (Fig. 3g). Although there was a clear trend for a higher *Firmicutes*:*Bacteroidetes* ratio in the WD control group relative to the LD control group, this difference did not rise to the level of statistical significance. On average, the *Firmicutes*:*Bacteroidetes* ratio for the WD control group was more than 2-fold greater than the groups that ended the study on LD.

### Genera

**Figure 4:**
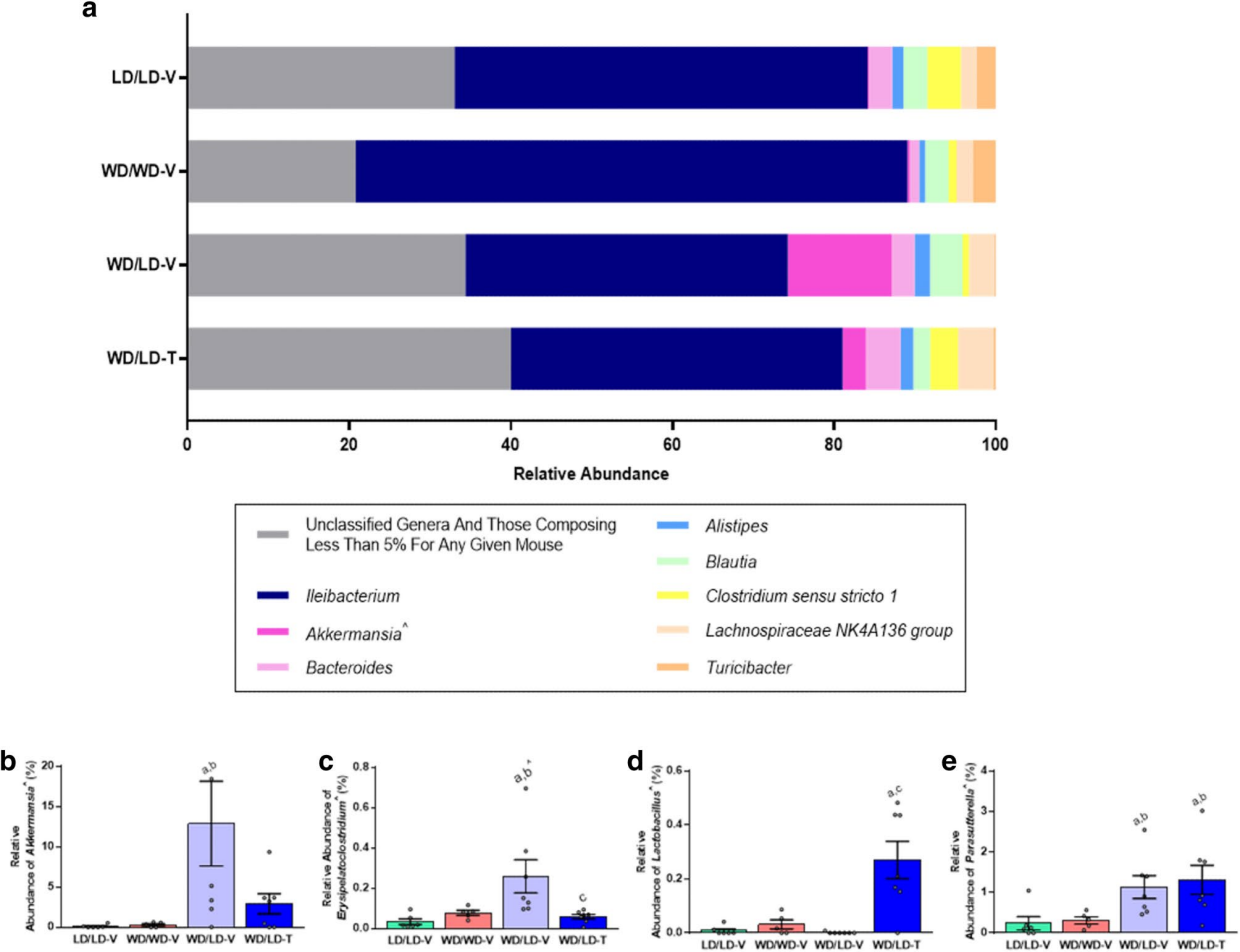
Microbial genera exhibited distinct effects of 5A-1MQ treatment and diet switch. (**a**) Relative abundance of the genera identified in the mouse cecal samples; sequences which could not be assigned (unclassified) were pooled with sequences composing less than 5% of any given mouse’s microbial genera only to simplify the graph. (**b–e**) Average relative abundance of the most interesting genera demonstrating significant differences between groups; the remaining genera can be found in Supplementary Figure S3. Graphs depict mean + /− SEM; unless specified, multiple comparisons were significant before and after FDR correction (*p* < 0.05 and q < 0.05); further statistical details in Supplementary Table S3. *a*, significantly different than the lean control group (LD/LD-V); *b*, significantly different than the obese control group (WD/WD-V); *c*, significantly different than vehicle-treated mice switched from WD to LD (WD/LD-V); ^p < 0.05 but q > 0.05.

Fifty-nine bacterial genera were definitively identified in the cecal samples. Sequences which could not be unambiguously assigned to a genus were labeled “unclassified” (Fig. 4a; Supplementary Table S3). The most abundant genus was *Ileibacterium*, a relatively novel genus of the *Firmicutes* phylum^48^. Consistent with the distribution of *Firmicutes* in the cecal samples, the relative abundance of *Ileibacterium* was highest in the WD controls (average relative abundance 68%), second highest in the LD controls (average relative abundance 51%), and lowest in the vehicle- and 5A-1MQ-treated mice switched from WD to LD (average relative abundances of 40% and 41%, respectively).

Significant main effects of treatment group (prior to Benjamini–Hochberg correction since none were significant after correction, although the necessity of correction for multiple comparisons in exploratory analyses remains unresolved^49^) were observed for the unclassified group and ten classified genera, namely, *Acetatifactor*, *Akkermansia*, *Erysipelatoclostridium*, [*Eubacterium*] *coprostanoligenes group*, *Family XIII UCG-001*, *Lactobacillus*, *Parasutterella*, *Ruminiclostridium 6*, *Ruminococcaceae UCG-004*, and *Ruminococcaceae UCG-009*. *Akkermansia* relative abundance was significantly higher in the vehicle-treated group switched from WD to LD compared to both the WD and LD control groups (Fig. 4b). *Erysipelatoclostridium* relative abundance was significantly greater in the vehicle-treated group switched from WD to LD relative to all other groups (before FDR correction, Fig. 4c). Uniquely, *Lactobacillus* relative abundance was dramatically higher in the 5A-1MQ-treated group switched from WD to LD compared to all other groups (Fig. 4d, statistically significantly different from LD controls and vehicle-treated mice switched from WD to LD). *Parasutterella* relative abundance was significantly higher in both the vehicle- and 5A-1MQ-treated groups switched from WD to LD relative to the LD and WD control groups (Fig. 4e). *Acetatifactor* relative abundance was greater in 5A-1MQ-treated mice switched from WD to LD compared to WD (without FDR correction) and LD controls (Supplementary Fig. S3). The relative abundance of the [*Eubacterium*] *coprostanoligenes group* was greater in both the vehicle- and 5A-1MQ-treated groups switched from WD to LD compared to the LD control group (Supplementary Fig. S3). Similarly, the relative abundance of *Family XIII UCG-001* was greater in the vehicle-treated (before FDR correction) and 5A-1MQ-treated groups switched from WD to LD compared to the LD control group (Supplementary Fig. S3).

Since *Ruminiclostridium 6* was only observed in the LD control group, it was unsurprising that its relative abundance was significantly greater in this group compared to all other experimental groups (Supplementary Fig. S3). *Ruminococcaceae UCG-004* was only present in the LD and WD control groups, with similar average relative abundances in both groups; however, the only statistically significant difference was between the LD control group and both groups switched from WD to LD, regardless of their treatment (Supplementary Fig. S3). In contrast, *Ruminococcaceae UCG-009* was present in all experimental groups, and exhibited significantly greater relative abundance in both the LD control group and the 5A-1MQ-treated group switched from WD to LD compared to the WD control group (both significant before FDR correction; Supplementary Fig. S3). Finally, the relative abundance of sequences that could not be assigned to a defined genus was significantly higher in vehicle- and 5A-1MQ-treated mice switched from WD to LD relative to the WD control group (the former before FDR correction only; Supplementary Fig. S3).

### Species

Several ASVs mapped with high confidence directly to a specific species (Supplementary Table S4), with considerably more species mapped using the BLASTn method compared to the BLCA method. Both methods generated the same species assignments for 60 ASVs, whereas 11 ASVs were mapped to dissimilar species by the different methods. Importantly, all ASVs within six of the genera mapped to identical species when analyzed by both methods. For *Erysipelatoclostridium,* every ASV mapped to [*Clostridium*] *cocleatum*. The only ASV identified as *Lactococcus* mapped to *Lactococcus lactis*. All ASVs for *Parasutterella* mapped to *Parasutterella excrementihominis*. Every ASV for *Romboutsia* mapped to *Romboutsia ilealis*; the only ASV identified for *Staphylococcus* mapped to *Staphylococcus xylosus*, and the only ASV found for *UBA1819* mapped to *Ruthenibacterium lactatiformans*. In six instances, ASVs that had not been assigned a genus by DECIPHER mapped consistently to a genera and species across the BLASTn and BLCA methods; the majority (i.e., four) of these ASVs mapped to *Dubosiella newyorkensis* while the remaining two ASVs mapped to *Romboutsia ilealis*.

### Linear discriminant analysis (LDA) effect size (LEfSe)

**Figure 5:**
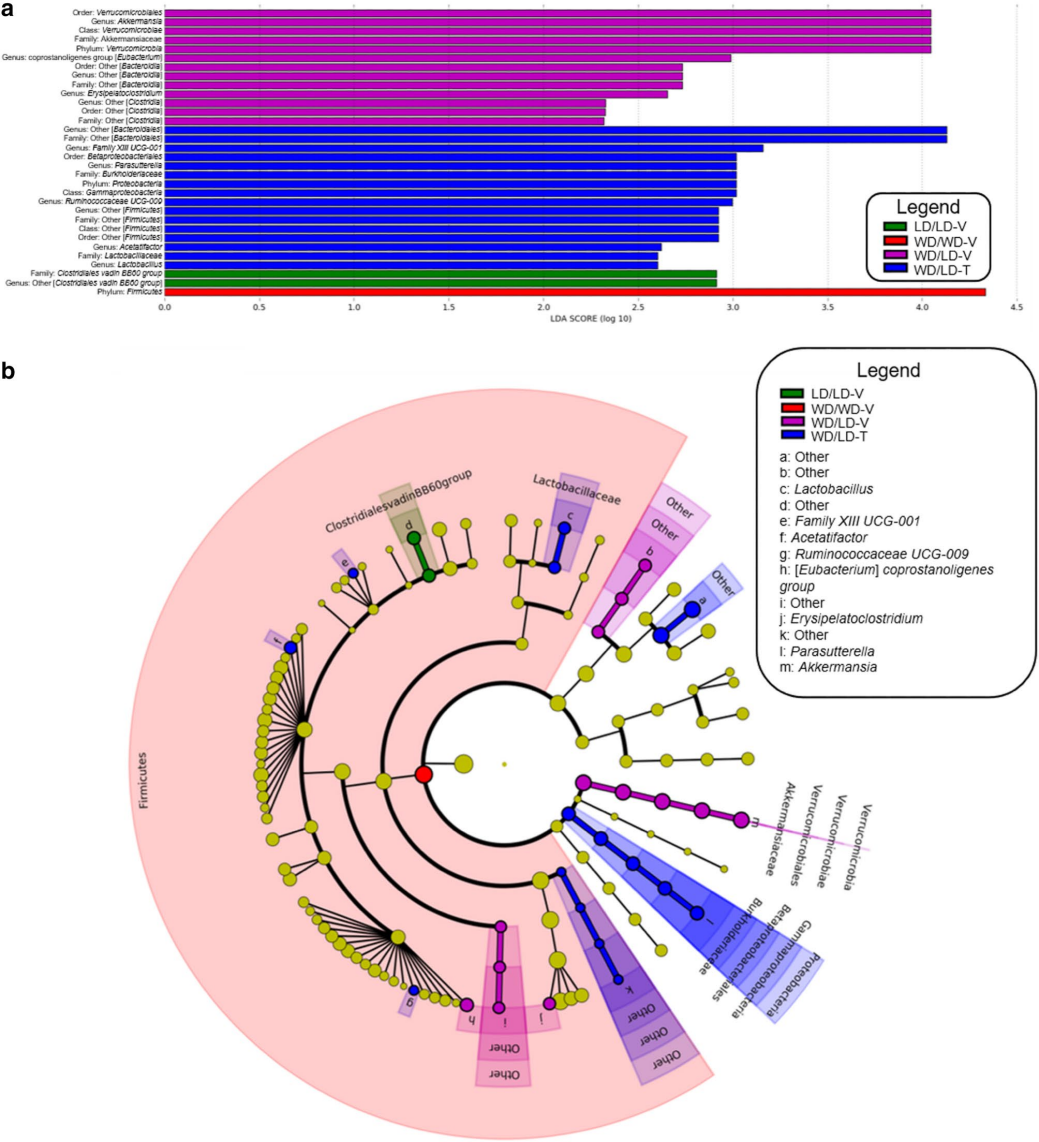
LEfSe strongly supported the results of the one-way ANOVA/Kruskal–Wallis tests. (**a**) Microbial classes identified in the LEfSe analysis as having a log_10_ LDA score > 2.0; each of the (non-“other”) phyla and genera identified by LEfSe was also identified as having a main effect (*p* < 0.05) of group in the one-way ANOVA/Kruskal–Wallis analyses. (**b**) The cladogram shows the relatedness of the phyla and genera findings; written labels begin with the highest class (phylum) in the outermost layer and end with the lowest class (genus) in the innermost layer. This figure was generated using the Huttenhower lab's Galaxy module (http://huttenhower.sph.harvard.edu/galaxy/) for LEfSe v1.0^102^.

LEfSe, which highlights biological consistency across subclasses^38^, identified (log_10_ LDA score > 2.0) many of the same genera and phyla noted as significant (*p* < 0.05) by one-way ANOVA/Kruskal–Wallis tests (i.e., the phyla *Verrucomicrobia*, *Proteobacteria*, and *Firmicutes*; the genera *Akkermansia*, [*Eubacterium*] *coprostanoligenes group*, *Erysipelatoclostridium*, and *Ruminococcaceae UCG-009).* These calculations (Fig. 5) attest to the excellent rigor and reproducibility of the one-way ANOVA/Kruskal–Wallis analyses of microbiome differences between treatment groups. While the majority of the analyses herein focused on the largest taxonomic rank below bacteria (phylum) and the smallest taxonomic rank this data could reasonably assign (genus), the LDA helped demonstrate how changes to the smallest rank resulted in dramatic shifts at the level of the largest rank below bacteria. Importantly, the LDA maintained the nearest taxonomic distinction, unlike the analyses that pooled together unclassified organisms.

Supporting the above data analyses, the LDA demonstrated that the *Verrucomicrobia*, *Proteobacteria*, and *Firmicutes* phyla had large effect sizes, suggesting that these particular phyla play a dominant role in differentiating the treatment groups from one another. The largest median was observed for *Verrucomicrobia* in the vehicle-treated group switched from WD to LD (WD/LD-V group), for *Proteobacteria* in the 5A-1MQ-treated group switched from WD to LD (WD/LD-T group), and for *Firmicutes* in the WD control (WD/WD-V) group (Fig. 5a). For classified genera (those not labeled “other” in the LEfSe analysis) in the microbiome, the largest effect sizes were observed in *Akkermansia*, [*Eubacterium*] *coprostanoligenes group*, *Erysipelatoclostridium*, *Family XIII-UCG-001*, *Parasutterella*, *Ruminococcaceae UCG-009*, *Acetatifactor*, and *Lactobacillus* (Fig. 5a), suggesting that the relative abundances in these genera are critical characteristics differentiating the treatment groups. *Akkermansia*, [*Eubacterium*] *coprostanoligenes group*, and *Erysipelatoclostridium* had the largest medians in the vehicle-treated group transitioned from WD to LD (WD/LD-V group), while *Family XIII-UCG-001*, *Parasutterella*, *Ruminococcaceae UCG-009*, *Acetatifactor*, and *Lactobacillus* had the largest medians in the 5A-1MQ-treated group transitioned from WD to LD (WD/LD-T group) (Fig. 5a).

When these LEfSe results were plotted as a cladogram (Fig. 5b), it became clearer that the treatment group with the largest median in a given class (e.g., genus) with a log_10_ LDA score > 2.0 was often consistent with the hierarchical class directly above it, if that class also had a log_10_ LDA score > 2.0. Additionally, the cladogram highlighted the large variety of microbes identified within the *Firmicutes* phyla, which was much greater than the microbe variation found in the other phyla. Finally, the cladogram (in addition to Supplementary Table S1) clearly illustrated that the unclassified genera fell within multiple orders/families; thus, analysis of pooled unclassified genera has severe limitations.

### K-means clustering

**Figure 6:**
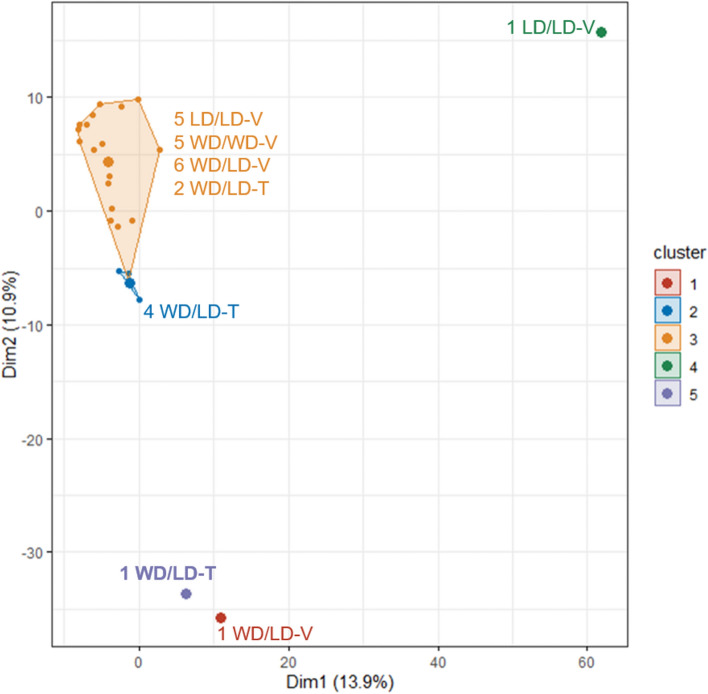
K-means clustering of the ASV relative abundance data by animal resulted in two primary clusters, one of which exclusively contained 5A-1MQ-treated mice transitioned from WD to LD (the WD/LD-T group). Cluster 1 contained only one vehicle-treated mouse transitioned from WD to LD (WD/LD-V). Cluster 2 contained four 5A-1MQ-treated mice transitioned from WD to LD (WD/LD-T), while Cluster 3 contained five LD control mice (LD/LD-V), five WD control mice (WD/WD-V), six WD/LD-V mice, and two WD/LD-T mice. Cluster 4 contained one LD/LD-V mouse and Cluster 5 contained one WD/LD-T mouse. Collectively, k-means clustering supported the conclusion that 5A-1MQ treatment elicits a unique ASV profile relative to the other treatment groups.

Five clusters were used for k-means clustering, per the results of the sum of squared estimate of errors curve, on the ASV relative abundance data with clustering by animal (n = 25). The two largest clusters contained 18 and 4 animals (Fig. 6). The latter cluster contained exclusively 5A-1MQ-treated animals switched from WD to LD (the WD/LD-T group), representing ~ 60% of all WD/LD-T animals. The other three treatment groups were represented approximately equally in the largest cluster, with a maximum of one animal per treatment group outside the cluster; however, the largest cluster included only two mice from the 5A-1MQ-treated group switched from WD to LD. The lack of substantial overlap between these two predominant clusters suggests that the WD/LD-T group has a distinct pattern of ASV relative abundances in its microbiome compared to the other treatment groups.

### Linkage between the cecal microbiome and adipose tissue metabolome

**Figure 7:**
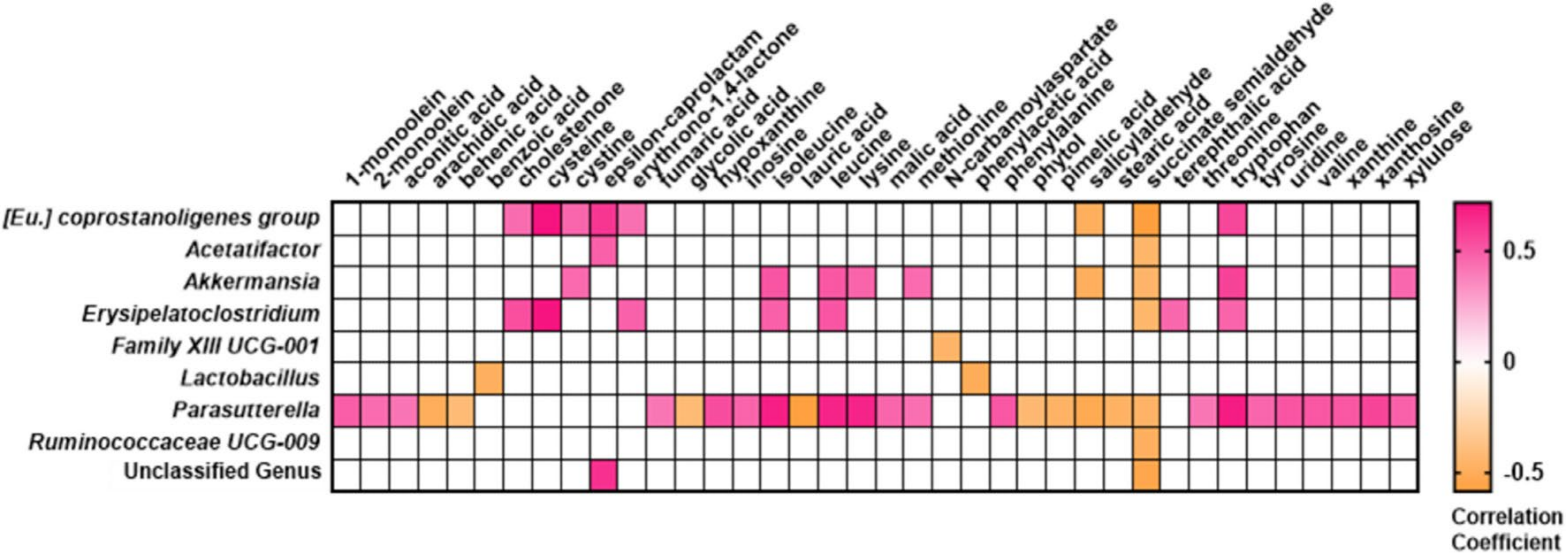
Correlations between the epididymal white adipose tissue metabolome and the microbiome strongly suggest that cecal *Parasutterella* abundance is linked to the abundance of many adipose metabolites. Significant correlations between genera and metabolites that demonstrated significant main effects of treatment group (significant prior to Benjamini–Hochberg correction) were used to generate a heat map colored by correlation coefficient. The data used to generate the heat map removed outliers by both metabolite and genus. Thus, the n varied per correlation. Correlations from the data including outliers can be found in Supplementary Table S5. *Eu.*, *Eubacterium.*

The relative abundance of a large number of microbial genera correlated with EWAT metabolite abundances. A total of 10,200 calculations were performed (in replicate, with and without outliers included) to identify potential links between microbiome genera and EWAT metabolites, and 439 genera-metabolite correlations were identified as statistically significant (*p* < 0.05, without Benjamini–Hochberg correction and with outliers excluded; Supplementary Table S5). A heat map of this data was generated using only the genera and metabolites that had previously demonstrated significant main effects of treatment group (Fig. 7); in total, 9 genera (including the unclassified group) and 38 metabolites met these criteria (metabolites published in Sampson et al., 2021^13^). Of note, the relative abundance of the *Parasutterella* genus, which was significantly enriched in both the vehicle- and 5A-1MQ-treated animals transitioned from WD to LD compared to both the WD and LD control groups, was observed to be significantly correlated (either positively or negatively) with 30 EWAT metabolites, both with and without outliers included.

## Discussion

Effective interventional treatments are critically needed to address ever-increasing rates of global obesity and obesity-linked comorbidities. NNMTi-treatment offers a promising therapeutic approach to address this unmet need^8,9,13^. There is growing evidence that the microbiome may be critical for establishing, maintaining, and reversing obesity^14–16^, and that both diet composition and adiposity each contribute to modulation of the mouse gut microbiome profile^49^. Consequently, it is important to understand how the microbiome responds to NNMTi-focused obesity treatments.

Multiple mechanisms may interrelate NNMTi treatment with the microbiome. Since NNMT is expressed in adipose, hepatic, and gut tissues^50–52^, and these tissues heavily cross-talk with the gut microbiome^53–55^, NNMTi treatment or its resulting weight loss effects^8,9^ may modulate the microbiome. Additionally, the NNMT substrate nicotinamide^12^ is metabolized by the microbiome^56,57^, and thus NNMTi may modulate substrate availability, potentially influencing microbial metabolic capacity to drive the NNMTi-mediated weight loss.

Given these potential mechanisms, this study was designed to test the hypothesis that DIO mice restored to the weight and adiposity of LD controls through NNMTi treatment and LD switch^13^ have a similar microbiome profile to mice continuously maintained on LD. Interestingly, we observed that the cecal microbiome of previously-obese animals returned to the lean state through NNMTi treatment and LD switch was distinct from the cecal microbiome of continuously lean control animals. Although we expected the largest microbiome differences to occur in the lean control group relative to the obese control group, we found that the diet-switched groups exhibited a greater number of differences from the control groups than the control groups did from one another. Importantly, this proof-of-concept study showed that the relative abundance of *Lactobacillus*, a genus associated with weight loss^58^, was uniquely increased and *Erysipelatoclostridium* was distinctly decreased in mice given 5A-1MQ treatment and an LD switch compared to mice switched to LD and given vehicle treatment. *Erysipelatoclostridium* is a member of the *Erysipelotrichaceae* family, which has been linked to obesity and related metabolic issues^59^.

**Figure 8:**
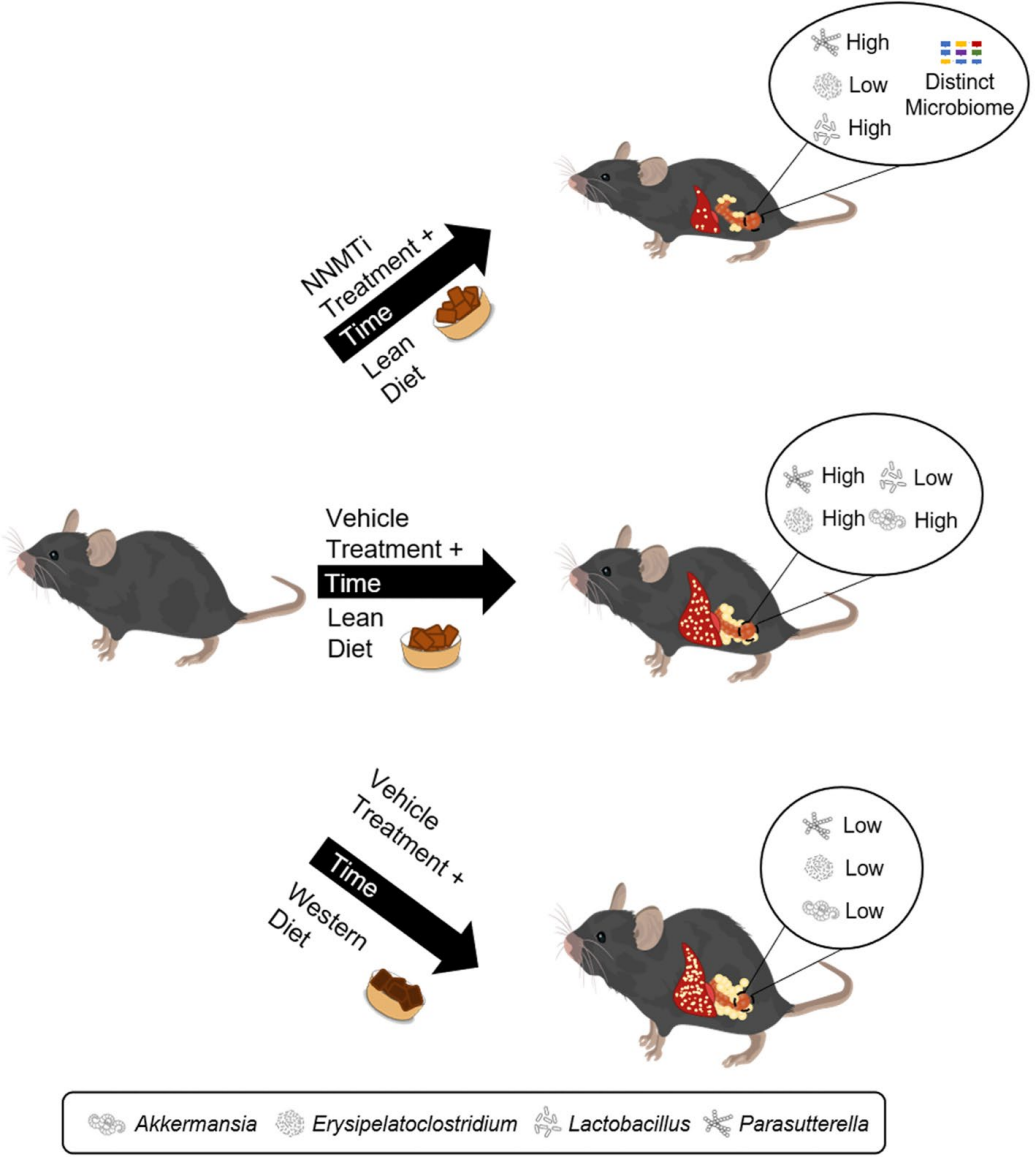
Key results summary. This figure was generated using a combination of open-source images, purchased Adobe stock photos, and PowerPoint.

The distinct microbiome profile of 5A-1MQ-treated mice was also supported by k-means clustering. Furthermore, the relative abundance of *Akkermansia*, a genus positively associated with improvements in obesity-related metabolic issues^60^, was increased in the vehicle-treated group that underwent diet switch relative to the WD control group, suggesting a potential for distinct microbiome-mediated mechanisms of weight loss subsequent to diet switch from those of diet switch in combination with 5A-1MQ treatment. However, the relative abundance of the genus *Parasutterella*, commonly decreased by HFD^61^, was increased in both the 5A-1MQ- and vehicle-treated mice switched from WD to LD, and the abundance of *Parasutterella* in all mice exhibited several correlations the EWAT metabolome. This may suggest that *Parasutterella* changes are linked to the diet switch or the series of diets the animals have experienced in their lifetime. A summary of these key results is found in Fig. 8 (graphic generated by ADW using creative commons- and Adobe-licensed images and PowerPoint).

In the present study, the cecal microbiome differed significantly across treatment groups, despite tightly controlling for several variables known to alter the microbiome in rodents. For example, parameters that have been shown in other studies to alter cecal and fecal microbiomes (e.g., vendor source^62,63^) were consistent across experimental groups in this study. Importantly, animals were singly housed to eliminate the exchange and cross-contamination of intestinal microbiomes, a factor that potentially biases studies with group-housed animals by minimizing microbiome variations for co-housed animals within a cohort. Unfortunately, singly housing is not commonly reported. For instance, the majority (58%) of mouse microbiome studies published in 2018–2019 did not report the number of mice per cage, and less than 20% of studies reported using singly-housed animals^64^. While singly-housed animals may experience stress that impacts metabolism, body weight, and adiposity^65–69^, the singly-housed animals in this study were treated identically (to the best of the experimenter’s ability) to minimize and equalize stress across all animals and reduce confounding variables.

Alpha diversity measures, calculated using several distinct methods, did not significantly differ between treatment groups. However, groups switched from WD to LD (WD/LD-V, WD/LD-T) tended to show greater alpha diversity compared to the control groups (WD/WD-V and LD/LD-V), which was not surprising since these animals experienced the most diet transitions with variable nutrient resources during their lifetime. Furthermore, this trend toward increased alpha diversity in the obese groups placed on an LD that had consequently experienced weight loss aligns with clinical observations, where low gene richness has been associated with increased adiposity, insulin resistance, and dyslipidemia^70^ while increased gene richness has been observed following dietary intervention in obese and overweight individuals^71^.

Singly-housing animals, which prevents inter-animal coprophagy, reduces beta diversity of the microbiome compared to stressful (chronic subordinate colony) co-housing conditions^72^. Furthermore, decreased bacterial richness has been linked to high carbohydrate^73^ and high-fat/low-protein concentrations^74^, as found in the lean and Western diets, respectively, the latter result only in old-aged rats^74^. However, irrespective of whether ASV abundance was weighted in the analysis, beta diversity analyses revealed distinct clustering by treatment group. The LD control group was particularly distinct from the other groups in the unweighted UniFrac and Bray–Curtis dissimilarity analyses’ plots, suggesting that animals exposed to the same diet at an early age in life diet develop similar microbiome profiles that persist even after an acute change in diet.

Microbiome differences were greater between the two groups switched from WD to LD (i.e., WD/LD-V and WD/LD-T) and the two diet control groups (i.e., LD/LD-V and WD/WD-V) compared to the microbiome differences observed between the lean (LD/LD-V) and obese (WD/WD-V) control groups. Importantly, the various diets contained identical fiber and protein content and varied primarily by the percentage of fat- and carbohydrate-derived kilocalories. Although dietary fiber has been associated with microbiome changes^75^, this is unlikely to account for the microbiome changes observed herein since fiber content was the same across diets. It is likely that the larger number of statistically significant changes to the microbiome observed in the groups switched from WD to LD reflects their more numerous exposures to different diets, longitudinal changes in dietary exposure, the stress associated with diet switch, the negative energy state achieved by treatment and/or diet-driven weight loss, or a combination of these factors, but it may also be related to differences in terminal body weight and fat mass since both adiposity and diet composition have been shown to modulate the mouse gut microbiome profile.

The finding of *Ileibacterium* as the most abundant cecal microbiome genus in every experimental group is relatively novel and may reflect experimental factors such as diet, housing conditions, and mouse strain. Mice from a different substrain and vendor, but fed similar lean and high-fat diets to this study, showed a predominance of the family *Erysipelotrichaceae,* of which *Ileibacterium* is a genus, in their cecal microbiomes^76^. Future studies using varied diets are needed to determine whether the predominance of *Ileibacterium* in the cecal microbiome holds pathological relevance.

One of the commonly reported microbiome differences between obese/HFD-fed animals and lean controls is an increased *Firmicutes*:*Bacteroidetes* ratio^43,44^. However, this ratio was not significantly different between this study’s obese and lean control groups. Several technical reasons may explain our distinctive findings, including differences in the microbiome sampling site, the limited n per group, singly housing mice, or the use of highly similar lean, Western, and high-fat diets which systematically exchanged fat content with carbohydrate content. Supporting a dominant role for diet composition in establishing the *Firmicutes*:*Bacteroidetes* ratio, previous work has shown that mice maintained on a high-fat diet had a similar cecal *Firmicutes*:*Bacteroidetes* ratio to those maintained on a high-carbohydrate diet^77^. This suggests that long-term maintenance on an LD rich in carbohydrates may confound some of the microbiome differences commonly reported between mice on HFDs and those on lean control diets which contain fewer carbohydrates.

Previous work has reported relatively increased *Firmicutes* and *Proteobacteria* and decreased *Bacteroidetes* in mice on an HFD, regardless of concomitant obesity (weight gain was minimized using a RELMβ knockout mouse^78^)^78,79^. These microbiome changes reversed rapidly, however, when the obese mice were returned to a normal, lean diet^79^. Similarly, in the current study, DIO mice from both groups that were switched from WD to LD exhibited higher *Bacteroidetes* and significantly lower *Firmicutes* relative abundances compared to DIO mice maintained on WD (WD controls; WD/WD-V). In contrast, DIO mice in both groups switched from WD to LD each had significantly increased *Proteobacteria* relative abundance (represented solely by the genus *Parasutterella*) compared to both the WD and the LD controls, suggesting that *Proteobacteria* relative abundance may be largely influenced by stress or the differences in net energy expenditure (or associated body weight changes) caused by the diet switch. Since decreased *Parasutterella* relative abundance may accompany increased stress^80,81^, the increased *Parasutterella* relative abundance that occurred in this study following diet switch likely arose from the weight loss-mediated negative energy state. Providing support for a causal link between a negative energy state and increased *Parasutterella* abundance, a rat model of obesity, which showed similar *Parasutterella* relative abundances in animals maintained on HFD and those maintained on normal chow but exhibited a treatment-induced increase in *Parasutterella* relative abundance, displayed a negative correlation between *Parasutterella* and body weight^82^. Our results also align with a previous observation that resveratrol treatment is associated with *Parasutterella* blooming in stool samples; both NNMT and resveratrol modulate Sirtuin 1 (Sirt1) activity^83,84^, and resveratrol^83^ and 5A-1MQ increase *Parasutterella* abundance, suggesting a potential causal link.

*Parasutterella* colonization has been shown to alter tryptophan, tyrosine, and xanthine metabolism in mouse cecal samples^61^. In this study, significant positive correlations were observed between tryptophan, tyrosine, and xanthine abundances in the EWAT and the relative abundance of *Parasutterella* in the cecal microbiome (Supplementary Table S5, outliers removed). Whether this observation is solely the consequence of translocation of *Parasutterella* bacteria from the intestine to the EWAT or driven by more indirect mechanisms such as bacterially-produced metabolites^85^ is yet to be determined. However, the large number of significant correlations noted in this study between *Parasutterella* relative abundance and EWAT metabolites strongly supports the relevance of this genus to the EWAT metabolome.

Groups switched from WD to LD had a greater average relative abundance of *Verrucomicrobia* (represented solely by the genus *Akkermansia*). In this genus, the relative abundance of the vehicle-treated group transitioned from WD to LD was significantly higher than both the lean and obese control groups. The *Akkermansia* species *muciniphila* has been shown to alter glucose and lipid metabolism as well as intestinal immunity^86^, to strengthen the integrity of the enterocyte monolayer to possibly improve intestinal barrier integrity^87^, and to improve glucose tolerance and reduce visceral adipose tissue inflammation in mice fed an HFD^88^. Cecal levels of *Akkermansia muciniphila* have been strongly correlated with physiological metrics including glycemia and insulinemia^89^, and *Akkermansia* has correlated negatively with obesity-related measures^90^ and has been implicated in DIO prevention^91,92^. 5A-1MQ-treated mice switched from WD to LD (WD/LD-T) showed a rather reduced relative abundance of *Akkermansia* compared to vehicle-treated DIO animals switched from WD to LD (WD/LD-V), suggesting a unique microbiome mechanism associated with weight loss in the vehicle-treated group switched from WD to LD compared to the 5A-1MQ-treated group that underwent the same diet switch.

5A-1MQ-treated mice switched from WD to LD were particularly distinct from mice in the other groups when k-means clustering was applied to the ASV relative abundance data. This result may have been driven by *Erysipelatoclostridium* and *Lactobacillus*, the *only* two microbial genera that significantly differed between the 5A-1MQ-treated and the vehicle-treated mice both switched from WD to LD. The relative abundance of *Erysipelatoclostridium* was increased in vehicle-treated mice that underwent the WD to LD switch (WD/LD-V) relative to the two control groups (LD/LD-V and WD/WD-V) but was not increased in 5A-1MQ-treated mice that underwent the same diet switch (WD/LD-T). Cecal levels of *Erysipelatoclostridium* abundance have been positively correlated with dyslipidemia^93^, and a report that WD decreased the relative abundance of *Erysipelatoclostridium* and that the abundance of *Erysipelatoclostridium* negatively correlated with obesity-related indices^90^ may partially explain our observation that decreased levels of *Erysipelatoclostridium* were present in the 5A-1MQ-treated group and the WD control group relative to the vehicle-treated group switched from WD to LD.

Unlike *Erysipelatoclostridium*, *Lactobacillus* was significantly and dramatically *increased* in 5A-1MQ-treated mice switched from WD to LD relative to their vehicle-treated counterparts and the LD control group. Several *Lactobacillus* species require nicotinamide or nicotinic acid for growth^94^; these metabolites are components of the NAD^+^ salvage pathway that are directly upstream of NNMT^12^. In addition, nicotinamide, the primary NNMT substrate^95,96^, is broken down by *Lactobacillus arabinosis* into nicotinic acid and ammonia^56^. Nicotinic acid is then assimilated into *Lactobacillus arabinosis* during growth^97^. We have previously demonstrated an increased nicotinamide:nicotinic acid ratio in the adipose tissues of 5A-1MQ-treated DIO mice^13^. NNMTi-mediated changes to the availability of nicotinamide/nicotinic acid in conjunction with the presence of moderate amounts of *Lactobacillus* may promote a positive feedback loop further increasing the abundance of *Lactobacillus*. Human studies using *Lactobacillus* as a probiotic have often demonstrated decreased body weight and/or body fat in a strain-dependent manner (for a review of human studies, see Crovesy et al., 2017^58^), suggesting that the NNMTi-and-LD-mediated changes to *Lactobacillus* relative abundance may be critical to NNMTi-mediated weight loss. *Lactobacillus*-mediated weight loss may be brought about, at least in part, by changes to lipid absorption and triglyceride synthesis, since orally-administered *Lactobacillus rhamnosus GG* reduces intestinal lipid absorption and the expression of triglyceride synthesis-related genes in mice^98^. Previous work has shown that the abundance of *Lactobacillus reuteri* (normalized to a control group) decreases during the onset of obesity and remains suppressed even after dieting in mice^99^, which parallels our observations that vehicle-treated diet-switched mice had lower levels of *Lactobacillus* than the 5A-1MQ-treated mice, the latter of which also demonstrated greater weight loss.

Although fundamental 5A-1MQ and LD-mediated changes to the microbiome were identified, this work is similar to many current studies where further investigations will clarify if changes in the microbiome are causative or associative^100^. Furthermore, this study’s robust design is not without limitations. Singly-housed rodents may have increased adiposity^101^ and, moreover, singly-housed mice in standard caging can engage in coprophagy of their own feces that may impact their cecal microbiome^102^. Changes to the microbial phyla and genera observed in the 5A-1MQ-treated group switched from WD to LD could have been driven by additive, synergistic, or independent mechanisms relative to those induced by diet switch alone, which could not be determined with the current study design that lacked a group treated with 5A-1MQ and maintained on WD. Since the first-line intervention for obesity is typically lifestyle change(s)^4^, this study purposefully combined LD switch with 5A-1MQ treatment; the effects of 5A-1MQ treatment on the microbiome of animals maintained continuously on LD or WD have yet to be determined. Additionally, the specificity of these results to the NNMTi 5A-1MQ and the dosage used herein also require further evaluation. Finally, although the correlations between the metabolome and microbiome were established using robust statistical analyses, there was limited statistical power and there are also limitations to the statistical approaches used herein (e.g., log transformation to resolve non-normal distribution and/or heteroscedasticity^103^). Nonetheless, this work has identified novel findings that support future studies to determine whether microbiome changes are contingent upon the combination of both NNMTi and LD switch and mediated through direct NNMTi-driven mechanisms or through indirect mechanisms such as weight or fat loss^8,13^.

## Supplementary Information

Supplementary Information 1.

Supplementary Figure 1.

Supplementary Figure 2.

Supplementary Figure 3.

Supplementary Figure 4.

Supplementary Table S1.

Supplementary Table S2.

Supplementary Table S3.

Supplementary Table S4.

Supplementary Table S5.

## Data Availability

The datasets generated and analyzed during this study are included in the Supplementary files.
