# Supplementary material for: Reduced calorie diet combined with NNMT inhibition establishes a distinct microbiome in DIO mice
Source: Sci Rep. 2022 Jan 10;12:484. doi: 10.1038/s41598-021-03670-5 (PMC8748953; doi:10.1038/s41598-021-03670-5)
Supplement: Supplementary file 1 — Supplementary Information 1. [file 41598_2021_3670_MOESM1_ESM.zip › ReadMe for Spearmans_Pearsons_Decider.docx]

The Spearman’s-Pearson’s Decider was developed in an effort to reduce the workload needed to use the proper statistical test for a correlational analysis. The parametric Pearson’s correlation has underlying assumptions which, although sometimes debated, require that 1) the data is acquired randomly and is representative of the population as a whole, 2) both variables are continuously measured 3) the variables have a bivariate normal distribution and their relationship is linear (which is somewhat assessed by the correlation itself), 4) there are not relevant outliers, and 5) the pairs for correlations are independent measures.^1^ Another common assumption, necessary for proper interpretation of r and r^2^ and sometimes considered the only necessary assumption, is that the variance is equal between the two variables measured, also called homoscedasticity of the data^2^. One common method to resolve non-normal distribution or heteroscedasticity (unequal variance between the two measurements) is log transformation^3^. The tool developed here begins by assessing whether data is normally distributed and homoscedastic, attempts to resolve non-normal distribution and/or heteroscedasticity using log transformation, and then runs the appropriate parametric (Pearson’s correlation) or non-parametric (Spearman’s correlation) accordingly. Groups without any variance are removed in an initial step. At present, the tool does not handle outliers, although robust outlier handling is a priority for future iterations.

1. Schober, Patrick MD, PhD, MMedStat; Boer, Christa PhD, MSc; Schwarte, Lothar A. MD, PhD, MBA. Correlation Coefficients: Appropriate Use and Interpretation, Anesthesia & Analgesia: May 2018 - Volume 126 - Issue 5 - p 1763-1768 doi: 10.1213/ANE.0000000000002864
2. Nefzger MD, Drasgow J. The needless assumption of normality in Pearson's r. American Psychologist. 1957;12(10):623–625. doi:10.1037/h0048216
3. Curran-Everett, Douglas. Explorations in Statistics: The Log Transformation: May 2018 – Volume 42 – Issue 2- p343-347 doi: 10.1152/advan.00018.2018
