# Supplementary figures and images for: Reduced calorie diet combined with NNMT inhibition establishes a distinct microbiome in DIO mice
Source: Sci Rep. 2022 Jan 10;12:484. doi: 10.1038/s41598-021-03670-5 (PMC8748953; doi:10.1038/s41598-021-03670-5)

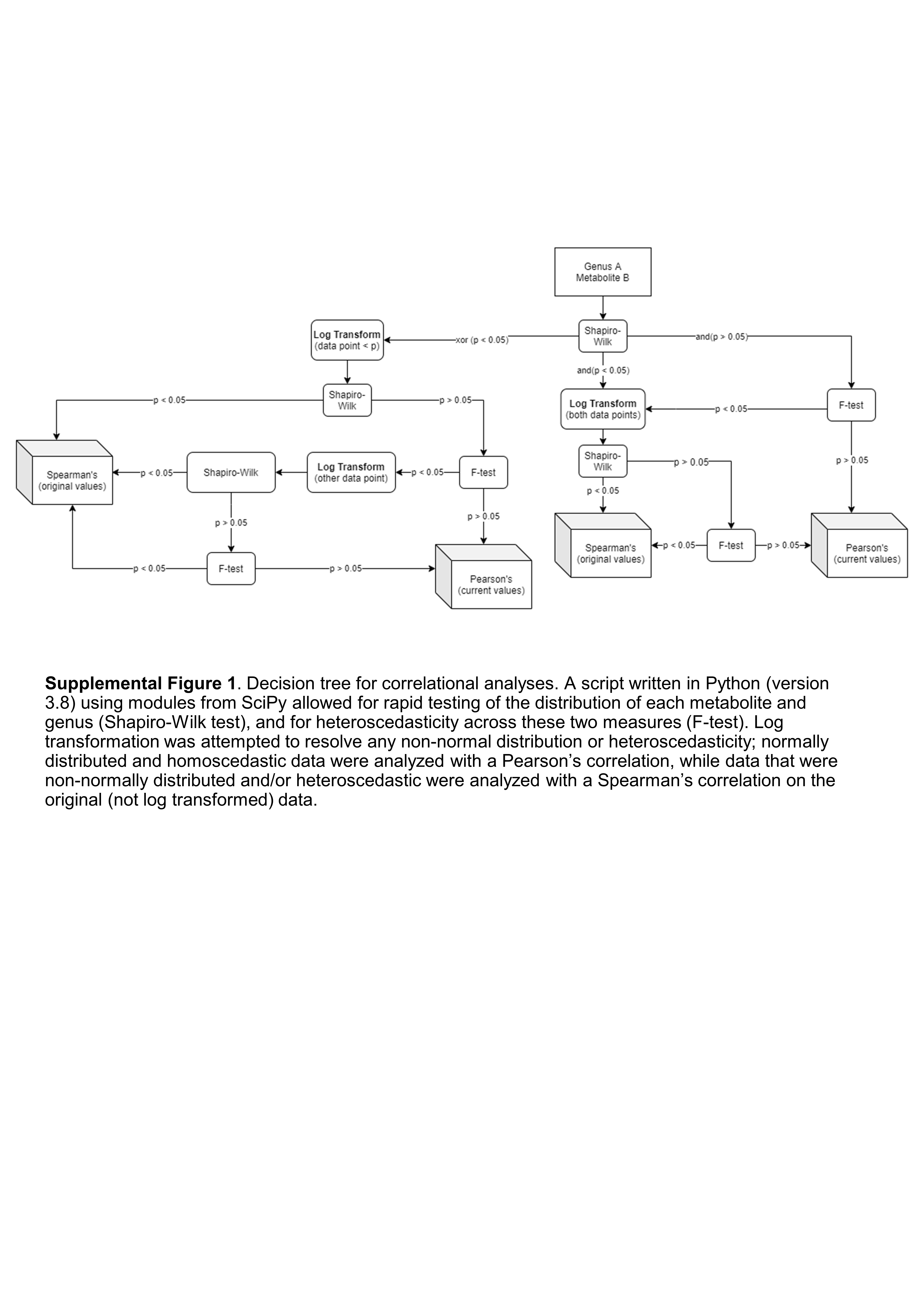

Supplement: Supplementary file 2 — Supplementary Figure 1. [file 41598_2021_3670_MOESM2_ESM.tif]

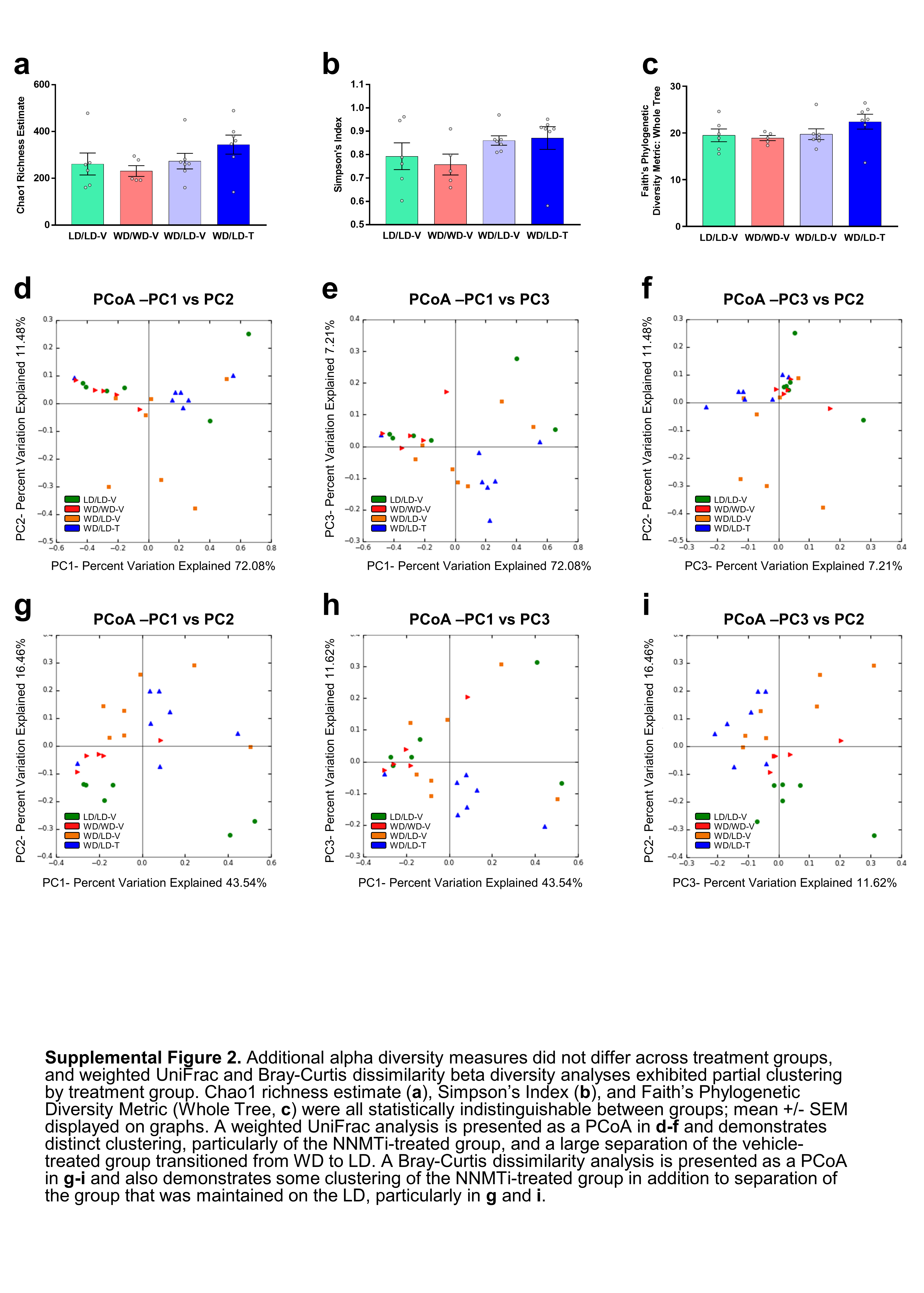

Supplement: Supplementary file 3 — Supplementary Figure 2. [file 41598_2021_3670_MOESM3_ESM.tif]

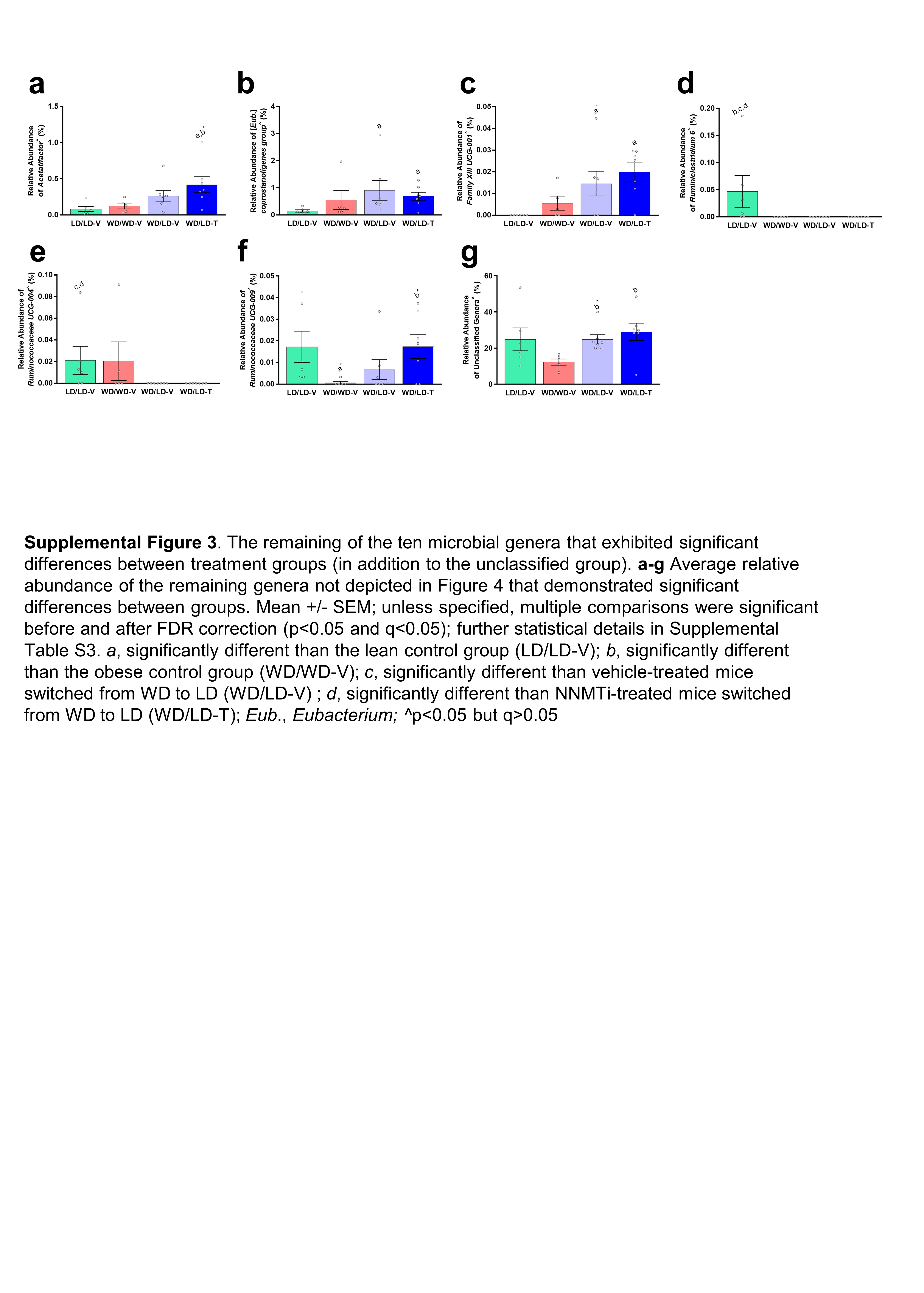

Supplement: Supplementary file 4 — Supplementary Figure 3. [file 41598_2021_3670_MOESM4_ESM.tif]

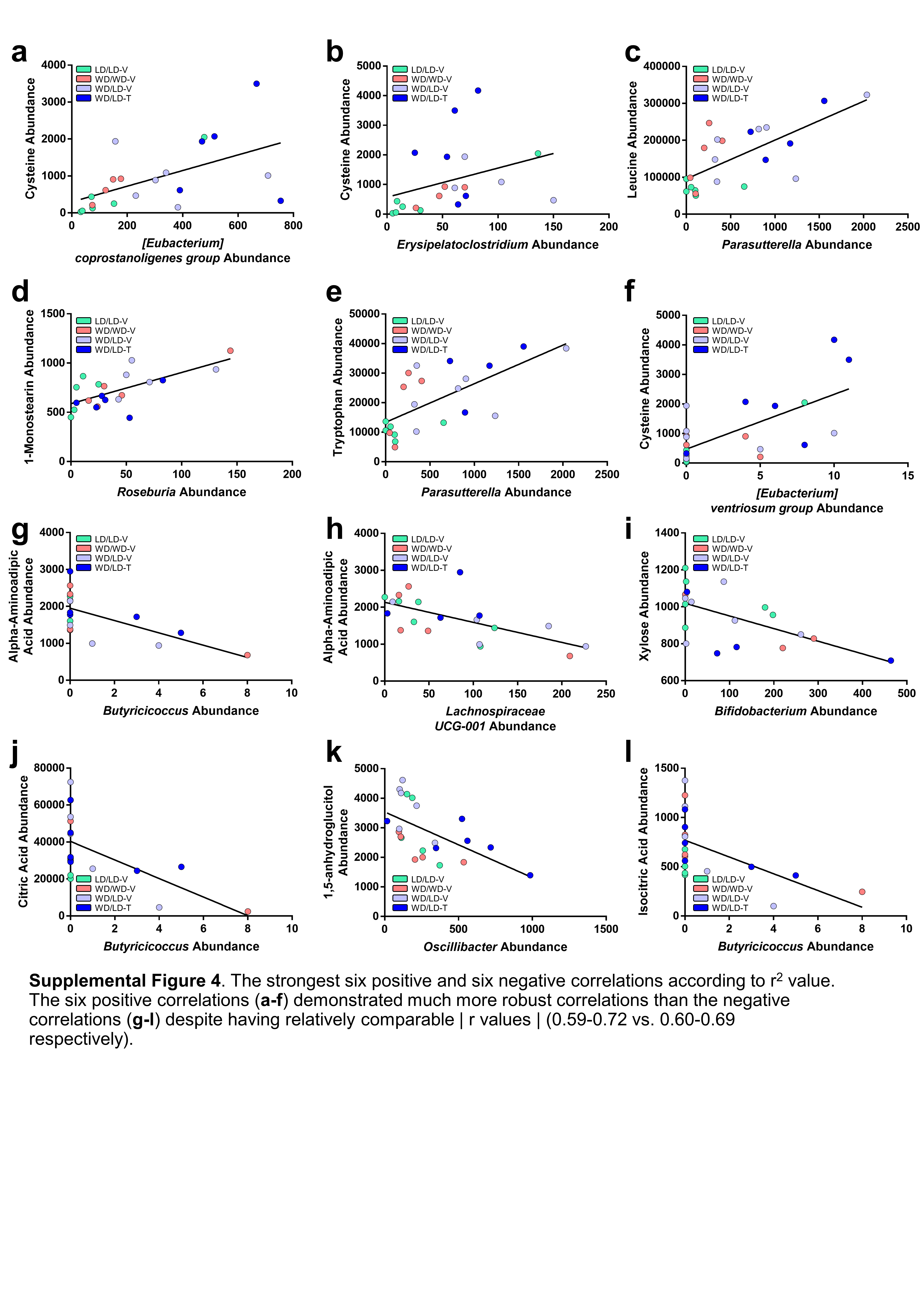

Supplement: Supplementary file 5 — Supplementary Figure 4. [file 41598_2021_3670_MOESM5_ESM.tif]
